# Phylogenetic analysis of the haemagglutinin gene of canine distemper virus strains detected from giant panda and raccoon dogs in China

**DOI:** 10.1186/1743-422X-10-109

**Published:** 2013-04-08

**Authors:** Ling Guo, Shao-lin Yang, Cheng-dong Wang, Rong Hou, Shi-jie Chen, Xiao-nong Yang, Jie Liu, Hai-bo Pan, Zhong-xiang Hao, Man-li Zhang, San-jie Cao, Qi-gui Yan

**Affiliations:** 1 College of Veterinary Medicine, Sichuan Agricultural University, Ya’an, China; 2 China Conservation and Research Center for the Giant Panda, Ya’an, China; 3 Chengdu Research Base for Giant Panda Breeding, Chengdu, China; 4 Enery-exit Inspection and Quarantine of Sichuan Province, Chengdu, China; 5 Southwest University for Nationalities, Chengdu, China; 6 Key laboratory of Animal Disease and Human Health of Sichuan Province, Sichuan Agricultural University, Ya’an, China

**Keywords:** Canine distemper virus, Haemagglutinin (H) gene, Genotype, Phylogenetic analysis

## Abstract

**Background:**

Canine distemper virus (CDV) infects a variety of carnivores, including wild and domestic *Canidae.* In this study, we sequenced and phylogenetic analyses of the hemagglutinin (H) genes from eight canine distemper virus (CDV) isolates obtained from seven raccoon dogs (*Nyctereutes procyonoides*) and a giant panda (*Ailuropoda melanoleuca*) in China.

**Results:**

Phylogenetic analysis of the partial hemagglutinin gene sequences showed close clustering for geographic lineages, clearly distinct from vaccine strains and other wild-type foreign CDV strains, all the CDV strains were characterized as Asia-1 genotype and were highly similar to each other (91.5-99.8% nt and 94.4-99.8% aa). The giant panda and raccoon dogs all were 549Y on the HA protein in this study, irrespective of the host species.

**Conclusions:**

These findings enhance our knowledge of the genetic characteristics of Chinese CDV isolates, and may facilitate the development of effective strategies for monitoring and controlling CDV for wild canids and non-cainds in China.

## Background

CD is caused by the canine distemper virus (CDV) which belongs to the Morbillivirus genus of the *Paramyxoviridae* virus family. This virus infects a broad range of animals, such as Mustelidae (ferrets, minks, skunks, weasels, and badgers), Procyonidae (raccoons), Ursidae (bears and pandas), Viverridae (civets, genets, and linsangs), hyaenidae (hyenas), and Felidae (lions and tigers) [1-7]. The genome of CDV encodes the following genes: matrix (M), fusion (F), hemagglutinin (H), nucleocapsid (N), polymerase (L), and phosphoprotein (P). The H gene protein is responsible for viral attachment to the cell host [8] and is the most variable protein described for all members of the genus Morbillivirus [9]. Experimental investigation showed that changing one amino acid (Y549H) in the CDV-H protein of one dog strain can decreased expression of specialist traits and increased expression of generalist traits, thereby confirming its functional importance [10].

The H gene has been used to investigate the genetic relationships among the various strains [11-13]. On the basis of the full-length sequence of the H gene of CDV strains identified globally, at least seven main geographic groups (genotypes) appear to circulate among the various susceptible hosts, namely America-1, America-2, Asia-1 and Asia-2, Europe, Europe wildlife and Arctic [14-17]. In addition, some CDV strains identified in Africa, Asia and Argentina [18-20] appear to diverge substantially and might represent separate geographic groups.

N glycosylation has been shown to be important for the correct folding, transport, and function of other *paramyxovirus* fusion and attachment glycoproteins [21]. There are significant molecular differences between the glycoproteins of wild-type and vaccine CDV strains. It has been hypothesized that reduced N glycosylation is an important attenuating factor and that an increase in N glycosylation may eventually result in vaccine failure [15,22].

Although the use of live attenuated vaccines has greatly helped to prevent CDV infections in susceptible animals, the dental abnormalities and enamel erosion that are found in many young and adult giant pandas [23,24] may be related to early infection with CDV [25,26]. Several episodes also have been reported in raccoon dogs [20]. So in this work we analyzed a total of 7 samples from raccoon dogs with clinical signs of canine distemper and 1 sample from giant panda which were submitted to our laboratories and the viruses were analyzed using a fragment of the H gene.

## Results

### CDV detection by NP-based RT-PCR

We analyzed a total number of 108 samples taken from raccoon dogs with suspect of CDV infection. CDV RNA was detected in 56 out of 108 (51.8%) blood samples, most of the raccoon dogs, between the age of 15–30 weeks. Negative samples were assayed twice to confirm the results. Forty-one of the 56 positive clinical specimens (73.2%) were obtained from raccoon dogs which had been vaccinated against CDV at least once. Twelve samples (21.4%) were obtained from unvaccinated raccoon dogs, and the vaccination status of the three remaining raccoon dogs was unknown. Forty-nine of the 56 positive animals (87.5%), showed symptoms typical of CDV, such as respiratory disease and neurological dysfunction, two raccoon dogs were asymptomatic, while no records were available for the other five raccoon dogs. The giant panda showed a mild fever (anal temperature 39.0°C).

### Sequence and phylogenetic analysis of the H gene

Amplification of fragments of the expected size was obtained with H gene and the sequences determined. After removing primer-derived sequences and some sequences with inconsistencies at the 5’ and 3’ ends, a 594 bp-long fragment of the H gene were obtained.

**Figure 1:**
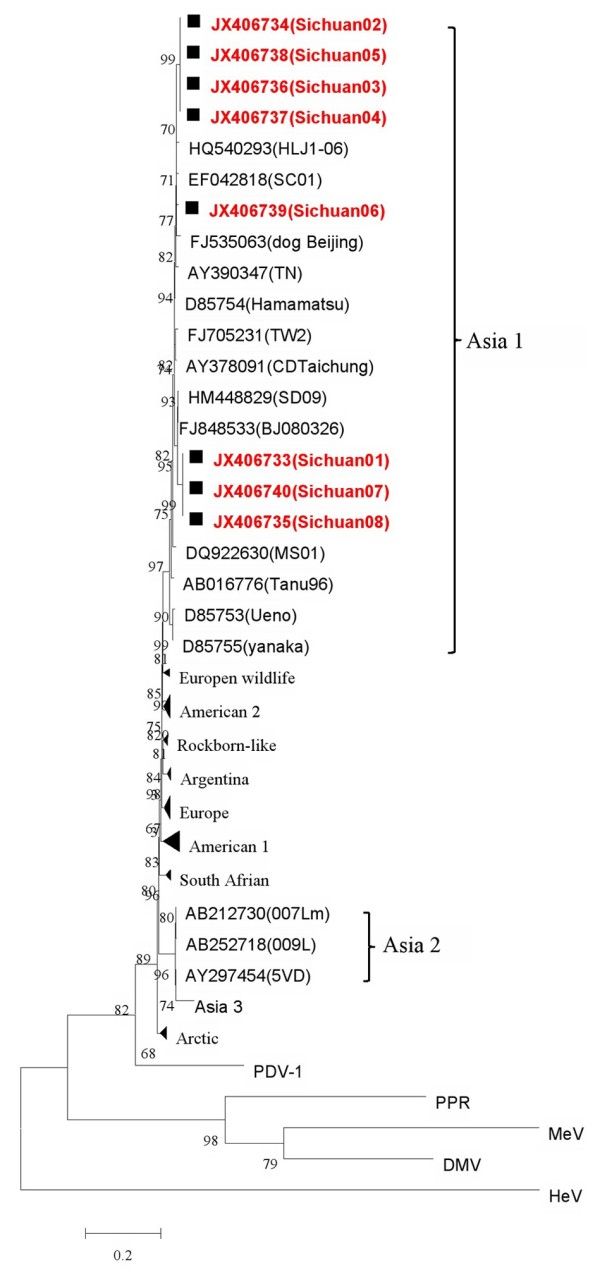
**Phylogenetic relationships among 73 CDV isolates based on the amino acid sequences of the H gene.** Distance values were calculated by the ClustalW program within the MEGA 5.0 software package. The Neighbor-Joining algorithm was used to generate the tree. Bootstrap values were calculated on 1000 replicates. Only bootstrap values >75% were shown. Filled quadrate (■) indicates the 8 Chinese wild-type CDV strains analyzed in this study.

The neighbor joining tree (Figure 1) generated using a 594 base pair (bp) long partial sequences of the H gene (amplified with primers “B”), evidenced geographic patterns (genotypes) resembling the patterns described previously using the full-length H gene (Asia 1, Asia 2, Europe, Arctic, America1 and America 2). In the phylogenetic tree (Figure 1) all the Chinese wild-type viruses (8/8) grouped together in one branch in the Asia-1 genotype. In this genotype, other Chinese CDV strains identified from domestic dogs or other wildlife species after the 1990s were also included, along with some Japanese and Korean strains detected before 1998. The 8 Asia-1 strains displayed identity to each other in the H gene (91.5-99.8% nt and 94.4-99.8% aa), while identity to the vaccine strains CDV3, Onderstepoort and Lederle was lower (85.4-89.2% nt and 84.4-88.3% at aa level) (Table 1). The 4 strains (Sichuan 02–05) displayed high identity to each other (97.4-99.8% nt and 98.4-99.8% aa). On the other hand, although strain Sichuan06 appeared to belong to the main Asia 1 clade, it is more distant from the group (Figure 1).

**Table 1:** Homology of nucleotide and amino acid sequences of CDV H gene

	**Sichuan01**	**Sichuan02**	**Sichuan03**	**Sichuan04**	**Sichuan05**	**Sichuan06**	**Sichuan07**	**Sichuan08**	**CDV3**	**Onderstepoot**	**Lederle**
	Nucleotides(%)										
Sichuan01		93.1	94.1	94.9	94.9	94.6	98.3	98.0	86.5	85.4	86.7
Sichuan02	94.4		98.5	98.3	98.3	96.6	94.9	94.9	89.1	87.7	89.2
Sichuan03	95.0	98.4		97.4	97.4	94.6	95.2	92.1	87.7	86.5	88.0
Sichuan04	95.0	99.4	99.7		99.8	97.3	97.5	91.7	88.0	86.5	88.2
Sichuan05	95.0	99.5	99.6	99.8		97.5	97.7	91.9	88.2	86.6	88.5
Sichuan06	95.5	98.9	99.4	99.5	99.4		95.9	91.5	88.0	86.5	88.2
Sichuan07	99.8	94.4	95.0	95.0	95.0	95.5		93.9	88.0	86.4	88.3
Sichuan08	99.7	94.5	95.3	95.0	95.0	95.5	99.5		88.4	87.2	88.6
CDV3	86.0	86.6	87.2	87.2	87.5	87.7	86.0	86.4		96.1	99.0
Onderstepoot	84.4	84.9	85.5	85.5	85.7	86.0	84.4	84.7	93.9		96.0
Lederle	86.6	87.2	87.7	87.7	87.9	88.3	86.6	86.8	97.2	93.9	
Amino acids(%)											

### Analysis of the amino acid sequences of the H gene of wild-type CDV strains

The inferred aa sequences of the partial H protein of the 8 wild-type Chinese CDV strains and of vaccine (America-1 genotype) strains were aligned. The giant panda and raccoon dogs all were 549Y on the HA protein from the current study (Table 2). Inspection of the H protein alignment (Table 3) revealed that all the 7 Asia-1 strains possessed 9 potential N-linked glycosylation sites (N-X-S/T) at aa positions 19–21, 149–151, 309–311, 391–393,422–424, 456–458, 584–586, 587–589 and 603–605. In the H protein of strain Sichuan01, the sites at positions 584–586 and 603–605 were disrupted. The strain Onderstepoort had only four glycosylation sites, while strains CDV3, Lederle and Convac displayed seven glycosylation sites.

**Table 2:** CDV isolates from China, organs from which they were isolated and their GenBank accession numbers

**Serial no**	**Isolate**	**Host**	**Foster mode**	**Vaccinated**	**Organ source**	**GenBank accession no.**	**549**
1	Sichuan01	Raccoon dog	Captivity	Yes	Blood	JX406733	Y
2	Sichuan02	Raccoon dog	Captivity	Yes	Blood	JX406734	Y
3	Sichuan03	Raccoon dog	Captivity	Yes	Blood	JX406736	Y
4	Sichuan04	Raccoon dog	Captivity	Yes	Blood	JX406737	Y
5	Sichuan05	Raccoon dog	Captivity	Yes	Blood	JX406738	Y
6	Sichuan06	Raccoon dog	Captivity	Yes	Blood	JX406739	Y
7	Sichuan07	Raccoon dog	Captivity	Yes	Blood	JX406740	Y
8	Sichuan08	Giant Panda	Captivity	Yes	Blood	JX406735	Y

**Table 3:** Distribution status of N-glycosylation sites of H gene in different genetypes of CDV

**Genetype**	**CDV isolate**	**N-glycosylation sites in each sequence**								
		**19**	**149**	**309**	**391**	**421**	**456**	**584**	**587**	**603**
Asia-1	Sichuan01	N	N	N	N	N	N	N	N	N
	Sichuan02	N	N	N	N	N	N	N	N	N
	Sichuan03	N	N	N	N	N	N	N	N	N
	Sichuan04	N	N	N	N	N	N	N	N	N
	Sichuan05	N	N	N	N	N	N	N	N	N
	Sichuan06	N	N	N	N	N	N	N	N	N
	Sichuan07	N	N	N	N	N	N	N	N	N
	Sichuan08	N	N	N	N	N	N	N	N	N
Vaccines	Onderstepoort	N	N	-	-	N	-	-	N	-
	Convac	N	N	-	N	N	N	-	N	N
	CDV3	N	N	-	N	N	N	-	N	N
	Lederle	N	N	-	N	N	N	-	N	N

Note:“N”: N-glycosylation Sites;“-”: negative.

## Discussion

Despite the vaccination procedures adopted in China in the last decades, CDV is still a serious threat to breeding raccoon dogs as well as to domestic dogs and it is responsible for relevant economic losses in Chinese fur industry [20]. In order to determine the frequency of this infection, we analyzed the samples taken from raccoon dogs with suspect of CDV infection. Our result revealed a relatively high (51.8%) prevalence of CDV infection animals sampled with clinical signs consistent with CDV infection. This indicated that CDV still represents a high risk to the canine population in China. Furthermore, all samples were tested twice and the results were reproducible. One possible explanation for the 52 animals thought to be infected with CDV were found negative by PCR screening is the ability of detecting low copy numbers of RNA. Another possible explanation for the negative is the presence of *Taq* polymerase inhibitors on blood samples, since it is known that blood material contains nucleases that degrades RNA, and substances, such as heparin and hemoglobin were found to inhibit the activity of *Taq* DNA polymerase [27].

The molecular analysis of the amplifications obtained by the use of primer pair “B” helped to clarify the relationship of the China strains with other CDV strains reported in other parts of the world. The analysis has shown that the strains of giant panda and raccoon dogs in this study are placed on one branches of the phylogenetic tree, were characterized as Asia-1 genotype. The H gene-based neighbor-joining phylogenetic tree analysis using selected sequences retrieved from GenBank revealed geographic-related patterns of segregation. Our findings are consistent with previous studies demonstrating the occurrence of strains belonging to genetically distinct CDV lineages in Asia [20,28,29]. The Chinese Asia-1 strains displayed a high genetic conservation with other Asia-1 CDVs, which detected from raccoon dogs or other carnivores in China over nearly two decades (Figure 1). The strain Sichuan08 was obtained from a mild fever giant panda in the Mar of 2012, and it showed the identity between Sichuan02-06 were 94.5-95.5%. Because there were few reports of giant panda clinical onset of CDV infection up to now, so we couldn't determine whether or not the fever is the clinical manifestations of the giant panda.

The observed diversity between the vaccine strains and recent wild-type CDVs may be accounted for by several mechanisms, such as adaptation to new host species [30,31], antigenic escape [32-35] and/or genetic recombination between wild-type strains [36], variously driving the evolution of the virus. It has been described that changes in N-glycosylation of the H protein may affect neutralization by antibodies and replication in vitro [15,37,38]. So, the connected aspartic amide N glycosylation site potentially is a spotlight in H proteins between vaccine and wild strains of CDV. Usually, there are four (Onderstepoort strain) or seven (CDV3, Lederle and Convac strain) potential sites in the vaccine strains. However, seven or nine sites have been detected in all wild CDV strains, of which 309–311 N-connected amide asparagine glycosylation sites are specific to CDV field strains. Some studies believed that the variants from H protein glycosylation played a crucial role in the antigenic differences and increase in N glycosylation may eventually result in vaccine failure [15,39]. So, we conjectured that the giant panda and raccoon dogs infected CDV maybe due to this reason in this time.

Based on phylogenetic analyses of complete sequences data from CDV strains retrieved from domestic dog and non-dog species it was hypothesized that positive selection drives residues 530 and 549 of the HA gene, and that the presence of specific residues at these positions resulted in the emergence of CDV as a disease of novel host species [31]. Recently, it has been suggested that there no evidence that amino acid 530 was strongly affected by host species, while wild canids there was a nearly significant trend towards a 549Y bias, non-canid strains showed no significant bias towards either H or Y at site 549 [9]. In this study, We found that amino acid 549Y was conserved within CDV lineages, regardless of host species. CDV transmission in wild carnivore and non-canids species may also most often occur between individuals within a species, and will be influenced by a range of factors such as population size and ranging patterns [40,41].

## Conclusions

In conclusion, the results presented by this study from a relatively small number of samples obtained from two host species conform to information obtained by other studies in China, that information currently available on CDV strains in China, including the results from the current study, is insufficient to determine whether or not the Asia 1 genotype is the predominant genotype in China. Analysis of CDV strains from a variety of host species will provide a more in-depth understanding of the global ecology of CDV, So, detect these more research on a broader range of potential carnivore hosts is required.

## Materials and methods

### Viruses and clinical specimens

#### Ethics statement

The animal from which specimens were collected, was handled in accordance with animal protection law of the People’s Republic of China (a draft of an animal protection law in China released on September 18, 2009). This study was approved by the National Institute of Animal Health Animal Care and Use Committee at Sichuan Agricultural University (approval number 2010–020).

From 2011 to 2012, 108 samples (whole blood) of raccoon dogs with suspect of CDV infection were sent to our laboratory for diagnostic confirmation and were identified only in 56 samples (51.8%) by using a RT-PCR, which amplifies a 242 bp-long fragment of the nucleoprotein (NP) gene with primers “A” ( Table 4). Out of 56 samples, 7 could be included in this study based on the amount of the sample and the amount of RNA extracts available for additional analysis. The samples were submitted to our laboratory from 3 different private fur farms within ya’an, Sichuan province. The organs of the giant panda (whole blood) were submitted to our laboratory by China Conservation and Research Center for the Giant Panda, Ya’an, China. Detailed information on the origin and the accession numbers of the CDV-positive samples is shown in Table 2.

**Table 4:** Description of the primer pairs

**Primer pair**	**Sequence 5’–3’**	**Target**	**Sense**	**Purpose**	**Amplicon size (bp)**
A	CGGAAATCAACGGACCTAAAT	N	+	Diagnostic	242
	TCCTTGAGCTTTCGACCCTT		_		
B	TGGTTCACAAGATGGTATTC	H	+	Phylogenetic	613
	CAACACCAC TAAATTGGACT		_		

### Extraction of nucleic acids

Viral RNA was extracted from leucocytes obtained by washing with nuclease-free water. Briefly, 500 μl of nuclease-free water were added to 500 μl of whole blood in a 1.5 microcentrifuge tube, and centrifuged 7 min at 5,000 g. A total of 500 μl of the supernatant was discarded, then 500 μl of nuclease-free water were added, repeating this step 3 times. Total RNA was extracted with TRI Reagent (Sigma®, USA), following the manufacturer’s instructions.

### RT-PCR

Reverse transcription was carried out by PrimeScript™ RT reagent Kit (TaKaRa, Dalian, China) according to the manufacturer’s instruction: 37°C for 15 min, 85°C for 5 sec, using 7 μl of RNA, 2 μl 5 × PrimeScript buffer, 0.5 μl random primer and 0.5 μl PrimeScript RT Enzyme. The CDV-positive samples were tested with primers “B” (Table 4), which amplifying a 613 bp fragment (corresponding to positions 8006–8619 of the Onderstepoort strain genome). The thermo-cycling condition were as follows: 5 min at 94°C (initial denaturation), 30 cycles of 30 sec at 94°C, 30 sec at 51°C, 30 sec at 72°C, and a final extension of 10 min at 72°C.

### Cloning and sequencing

PCR products of the correct size (613 bp in length) were amplified and cloned into the pMD 19-T vector (TaKaRa, Dalian, China). For each CDV strain, 3–5 positive recombinant plasmids were sequenced in both directions using primer M13 (Shanghai Invitrogen Biotechnology Company, Shanghai, China) and additional primers selected on the basis of the obtained sequences. The accession numbers are shown in Table 2.

### Amino acid analysis

To investigate the differences between the Chinese wild-type CDV strains and the vaccine strains available in the market in China, the deduced amino acid (aa) sequences of the H protein were aligned. Multiple sequence alignment was carried out using the software package MegAlign (Lasergene7.0, DNAStar Inc., Madison, WI). The aa present at sites 549 on the HA protein was determined for the eight strains from the current study. The potential N-linked glycosylation sites of the H protein were determined using the software NetNGlyc1.0.

### Phylogenetic analysis

The eight China CDV sequences were compared to those of 64 CDV strains that were obtained from various part of the world up until November 2010. The Accession numbers for these H gene nucleotide sequences were obtained from the GenBank nucleotide database at the National Center for Biotechnology Information (NCBI). The nucleotide sequences were analyzed preliminary with cognate sequences available in GenBank using BLAST software (http://www.ncbi.nlm.nih.gov/). Multiple sequence alignment was made with ClustalW software [42] included in MEGA 5.0 software [43].

Sequences for comparison were obtained corresponded to nucleotide sequences of the H gene. Phylogenetic analysis of nucleotide were computed using the Kimura two parameter algorithm and the tree was constructed with the Neighbor Joining method with a 1000 bootstrap repeats using the MEGA 5.0 Software [43]. The measles virus (MeV) (NC001498), phocine (seal) distemper virus (PDV) (AF479274), peste-des- petits-ruminants virus (PPRV) (NC006383), hendra virus (HeV) (NC001906) and dolphin morbillivirus (DMV) (NC005283) were used as outgroup in the neighbor joining trees.

## Competing interests

The authors declare that they have no competing interests.

## Authors’ contributions

Conceived and designed the experiments: LG QGY SJC. Performed the experiments: LG SLY ZXH JL HBP. Analyzed the data: LG SLY MLZ. Contributed reagents/materials /analysis tools: SJC CDW RH XNY . Wrote the paper: LG SLY. All of the authors read and approved the final version of the manuscript.

## References

[B1] Appel MJG Yates RA Foley GL Bernstein JJ Santinelli S Spelman LH Miller LD Arp LH Anderson M Barr M Canine distemper epizootic in lions, tigers, and leopards in North America J Vet Diagn Invest 1994 6 277 10.1177/104063879400600301 7948195

[B2] Deem SL Spelman LH Yates RA Montali RJ Canine distemper in terrestrial carnivores: a review J Zoo Wildlife Med 2000 31 441 451 10.1638/1042-7260(2000)031[0441:CDITCA]2.0.CO;2 11428391

[B3] Evermann J Leathers C Gorham J McKeirnan A Appel M Pathogenesis of two strains of lion (Panthera leo) morbillivirus in ferrets (Mustela putorius furo) Vet Pathol 2001 38 311 10.1354/vp.38-3-311 11355661

[B4] Pearson R Gorham J Canine distemper virus Virus 1987 1 371 373

[B5] Van De Bildt MWG Kuiken T Visee AM Lema S Fitzjohn TR Osterhaus ADME Distemper outbreak and its effect on African wild dog conservation Emerg Infect Dis 2002 8 212 10.3201/eid0802.010314 PMC2732438 11897078

[B6] Appel MJ Summers BA Pathogenicity of morbilliviruses for terrestrial carnivores Book Pathogenicity of morbilliviruses for terrestrial carnivores 1995 4 187 191 10.1016/0378-1135(95)00011-x 8588312

[B7] Haas L Hofer H East M Wohlsein P Liess B Barrett T Canine distemper virus infection in Serengeti spotted hyenas Vet Microbiol 1996 48 147 152 8861651 10.1016/0378-1135(95)00180-8

[B8] Von Messling V Zimmer G Herrler G Haas L Cattaneo R The hemagglutinin of canine distemper virus determines tropism and cytopathogenicity J Virol 2001 75 6418 6427 10.1128/JVI.75.14.6418-6427.2001 11413309 PMC114365

[B9] Nikolin VM Wibbelt G Michler FU Wolf P East ML Susceptibility of carnivore hosts to strains of canine distemper virus from distinct genetic lineages Vet Microbiol 2012 156 45 53 10.1016/j.vetmic.2011.10.009 22024346

[B10] Nikolin VM Osterrieder K von Messling V Hofer H Anderson D Dubovi E Brunner E East ML Antagonistic pleiotropy and fitness trade-offs reveal specialist and generalist traits in strains of canine distemper virus PLoS One 2012 7 e50955 10.1371/journal.pone.0050955 23239996 PMC3519774

[B11] Demeter Z Lakatos B Palade EA Kozma T Forgách P Rusvai M Genetic diversity of Hungarian canine distemper virus strains Vet Microbiol 2007 122 258 269 10.1016/j.vetmic.2007.02.001 17350769 PMC7117499

[B12] Haas L Martens W Greiser-Wilke I Mamaev L Butina T Maack D Barrett T Analysis of the haemagglutinin gene of current wild-type canine distemper virus isolates from Germany Virus Res 1997 48 165 171 10.1016/S0168-1702(97)01449-4 9175255

[B13] Martella V Cirone F Elia G Lorusso E Decaro N Campolo M Desario C Lucente M Bellacicco A Blixenkrone-Møller M Heterogeneity within the hemagglutinin genes of canine distemper virus (CDV) strains detected in Italy Vet Microbiol 2006 116 301 309 10.1016/j.vetmic.2006.04.019 16730927

[B14] Bolt G Jensen TD Gottschalck E Arctander P Appel MJ Buckland R Blixenkrone-Moller M Genetic diversity of the attachment (H) protein gene of current field isolates of canine distemper virus J Gen Virol 1997 78 367 372 9018059 10.1099/0022-1317-78-2-367

[B15] Iwatsuki K Miyashita N Yoshida E Gemma T Shin YS Mori T Hirayama N Kai C Mikami T Molecular and phylogenetic analyses of the haemagglutinin (H) proteins of field isolates of canine distemper virus from naturally infected dogs J Gen Virol 1997 78 373 380 9018060 10.1099/0022-1317-78-2-373

[B16] Martella V Elia G Lucente MS Decaro N Lorusso E Banyai K Blixenkrone-Møller M Lan NT Yamaguchi R Cirone F Genotyping canine distemper virus (CDV) by a hemi-nested multiplex PCR provides a rapid approach for investigation of CDV outbreaks Vet Microbiol 2007 122 32 42 10.1016/j.vetmic.2007.01.005 17275219

[B17] Pardo ID Johnson GC Kleiboeker SB Phylogenetic characterization of canine distemper viruses detected in naturally infected dogs in North America J Clin Microbiol 2005 43 5009 5017 10.1128/JCM.43.10.5009-5017.2005 16207955 PMC1248462

[B18] Calderon MG Remorini P Periolo O Iglesias M Mattion N La Torre J Detection by RT-PCR and genetic characterization of canine distemper virus from vaccinated and non-vaccinated dogs in Argentina Vet Microbiol 2007 125 341 349 10.1016/j.vetmic.2007.05.020 17628358

[B19] Woma TY van Vuuren M Bosman AM Quan M Oosthuizen M Phylogenetic analysis of the haemagglutinin gene of current wild-type canine distemper viruses from South Africa: lineage Africa Vet Microbiol 2010 143 126 132 10.1016/j.vetmic.2009.11.013 20060661

[B20] Zhao JJ Yan XJ Chai XL Martella V Luo GL Zhang HL Gao H Liu YX Bai X Zhang L Phylogenetic analysis of the haemagglutinin gene of canine distemper virus strains detected from breeding foxes, raccoon dogs and minks in China Vet Microbiol 2010 140 34 42 10.1016/j.vetmic.2009.07.010 19647380

[B21] Hu A Cattaneo R Schwartz S Norrby E Role of N-linked oligosaccharide chains in the processing and antigenicity of measles virus haemagglutinin protein J Gen Virol 1994 75 1043 1052 10.1099/0022-1317-75-5-1043 8176366

[B22] Sawatsky B von Messling V Canine distemper viruses expressing a hemagglutinin without N-glycans lose virulence but retain immunosuppression J Virol 2010 84 2753 2761 10.1128/JVI.01813-09 20042514 PMC2826070

[B23] Janssen DL Edwards MS Sutherland-Smith M Yu J Li D Zhong G Wei R Chenglin Z Miller R Phillips L Significant medical issues and biological reference values for giant pandas from the biomedical survey Giant pandas: biology, veterinary medicine, and management 2006 Cambridge, U.K: Cambridge University Press 59 86

[B24] Janssen DL Morris P Sutherland-Smith M Greenberg M Li D Hu D Mauroo N Spelman L Medical management of captive adult and geriatric giant pandas Giant pandas: biology, veterinary medicine, and management 2006 Cambridge, U.K: Cambridge University Press 353 376

[B25] Bittegeko S Arnbjerg J Nkya R Tevik A Multiple dental developmental abnormalities following canine distemper infection J Am Anim Hosp Assoc 1995 31 42 45 7820764 10.5326/15473317-31-1-42

[B26] Dubielzig RR Higgins RJ Krakowka S Lesions of the enamel organ of developing dog teeth following experimental inoculation of gnotobiotic puppies with canine distemper virus Vet Pathol 1981 18 684 689 7281465 10.1177/030098588101800513

[B27] De Paula SO Lopes da Fonseca BA Optimizing dengue diagnosis by RT-PCR in IgM-positive samples: comparison of whole blood, buffy-coat and serum as clinical samples J Virol Methods 2002 102 113 117 10.1016/S0166-0934(02)00005-8 11879699

[B28] Wang F Yan X Chai X Zhang H Zhao J Wen Y Wu W Differentiation of canine distemper virus isolates in fur animals from various vaccine strains by reverse transcription-polymerase chain reaction-restriction fragment length polymorphism according to phylogenetic relations in china J Virol 2011 8 85 10.1186/1743-422X-8-85 PMC3056815 21352564

[B29] Tan B Wen Y-J Wang F-X Zhang S-Q Wang X-D Hu J-X Shi X-C Yang B-C Chen L-Z Cheng S-P Pathogenesis and phylogenetic analyses of canine distemper virus strain ZJ7 isolate from domestic dogs in China J Virol 2011 8 520 10.1186/1743-422X-8-520 PMC3229531 22087872

[B30] Domingo E Holland JJ RNA virus mutations and fitness for survival Annu Rev Microbiol 1997 51 151 178 10.1146/annurev.micro.51.1.151 9343347

[B31] McCarthy AJ Shaw MA Goodman SJ Pathogen evolution and disease emergence in carnivores Proc Biol Sci 2007 274 3165 3174 10.1098/rspb.2007.0884 17956850 PMC2293938

[B32] Iwatsuki K Tokiyoshi S Hirayama N Nakamura K Ohashi K Wakasa C Mikami T Kai C Antigenic differences in the H proteins of canine distemper viruses Vet Microbiol 2000 71 281 286 10.1016/S0378-1135(99)00172-8 10703710

[B33] Lan NT Yamaguchi R Furuya Y Inomata A Ngamkala S Naganobu K Kai K Mochizuki M Kobayashi Y Uchida K Tateyama S Pathogenesis and phylogenetic analyses of canine distemper virus strain 007Lm, a new isolate in dogs Vet Microbiol 2005 110 197 207 10.1016/j.vetmic.2005.07.016 16144749

[B34] Lan NT Yamaguchi R Inomata A Furuya Y Uchida K Sugano S Tateyama S Comparative analyses of canine distemper viral isolates from clinical cases of canine distemper in vaccinated dogs Vet Microbiol 2006 115 32 42 10.1016/j.vetmic.2006.01.010 16504421

[B35] Martella V Elia G Lucente MS Decaro N Lorusso E Banyai K Blixenkrone-Moller M Lan NT Yamaguchi R Cirone F Genotyping canine distemper virus (CDV) by a hemi-nested multiplex PCR provides a rapid approach for investigation of CDV outbreaks Vet Microbiol 2007 122 32 42 10.1016/j.vetmic.2007.01.005 17275219

[B36] Han GZ Liu XP Li SS Cross-species recombination in the haemagglutinin gene of canine distemper virus Virus Res 2008 136 198 201 10.1016/j.virusres.2008.04.022 18550189

[B37] Harder TC Kenter M Vos H Siebelink K Huisman W van Amerongen G Orvell C Barrett T Appel MJ Osterhaus AD Canine distemper virus from diseased large felids: biological properties and phylogenetic relationships J Gen Virol 1996 77 397 405 10.1099/0022-1317-77-3-397 8601773

[B38] Lan NT Yamaguchi R Kawabata A Uchida K Sugano S Tateyama S Comparison of molecular and growth properties for two different canine distemper virus clusters, Asia 1 and 2, in Japan J Vet Med Sci 2007 69 739 744 10.1292/jvms.69.739 17675806

[B39] Zhao J Yan X Wu W Genetic variations and cellular receptors of Canine distemper virus–a review Acta Microbiol Sin 2008 48 986 18837382

[B40] Guiserix M Bahi-Jaber N Fouchet D Sauvage F Pontier D The canine distemper epidemic in Serengeti: are lions victims of a new highly virulent canine distemper virus strain, or is pathogen circulation stochasticity to blame? J R Soc Interface 2007 4 1127 1134 10.1098/rsif.2007.0235 17456450 PMC2396208

[B41] Begon M Townsend CR Harper JL Ecology: From Individuals to Ecosystems 2006 Oxford, UK: Blackwell

[B42] Thompson JD Higgins DG Gibson TJ CLUSTAL W: improving the sensitivity of progressive multiple sequence alignment through sequence weighting, position-specific gap penalties and weight matrix choice Nucleic Acids Res 1994 22 4673 4680 10.1093/nar/22.22.4673 7984417 PMC308517

[B43] Tamura K Peterson D Peterson N Stecher G Nei M Kumar S MEGA5: molecular evolutionary genetics analysis using maximum likelihood, evolutionary distance, and maximum parsimony methods Mol Biol Evol 2011 28 2731 2739 10.1093/molbev/msr121 21546353 PMC3203626

